# Proteomic signatures of equine dental tooth tissues in ageing and disease

**DOI:** 10.1002/evj.70167

**Published:** 2026-05-15

**Authors:** Anders Jensen, Danae Emilie Zambouli, Iris Gringel, Zoe Nugent, Kazuhiro Yamamoto, Lee Cooper, Andrew J. Peffers, Guido Rocchigiani, Mandy J. Peffers

**Affiliations:** ^1^ Department of Musculoskeletal and Ageing Science, Institute of Life Course and Medical Sciences University of Liverpool Liverpool UK; ^2^ Department of Clinical Sciences School of Veterinary Medicine, Aristotle University of Thessaloniki Thessaloniki Greece; ^3^ Institute of Life Course and Medical Sciences, School of Dentistry University of Liverpool Liverpool UK; ^4^ North Wales Equine Dental Practice, Bryn Common Ffrith UK; ^5^ Institute of Infection, Veterinary and Ecological Sciences University of Liverpool, Leahurst Campus Neston UK

**Keywords:** cementum, dentin, enamel, horse, proteomics, pulp

## Abstract

**Background:**

Ageing and dental disease in horses lead to structural and functional deterioration of dental tissues, yet their molecular signatures remain poorly characterised. Understanding how these processes alter the protein composition of enamel, dentin, cementum and pulp is essential for improving equine oral health and identifying biomarkers of degeneration.

**Objectives:**

To characterise the proteomic profiles of equine dental tissues and determine age‐ and disease‐associated changes, with the aim of identifying proteins consistently altered across conditions.

**Study Design:**

Ex vivo proteomic study.

**Methods:**

Fifteen equine cheek teeth were allocated to three groups (*n* = 5/group): mature adult, old and diseased. Label‐free quantitative mass spectrometry was used to profile proteins across enamel, dentin, cementum and pulp. Differential abundance analysis and clustering assessed age‐ and disease‐related effects. A Shiny‐based interactive tool was developed for data exploration.

**Results:**

A total of 1950 proteins were identified, with 58% unique to dental pulp. Ageing affected 329 proteins across all tissues, while disease impacted 379. Ageing consistently disrupted extracellular matrix (ECM) homeostasis in hard tissues. Cementum demonstrated increased catabolic markers the metalloproteinases −2 and −13. In dentin, the mineralisation protein dentin sialophosphoprotein was significantly reduced with age. Altered proteins in diseased pulp demonstrated predicted inhibition of ECM organisation (*z*‐score = −3.05, *p* = 0.032) and elastic fibre formation (*z*‐score = −2.82, *p* = 0.0033), while retinoic acid receptor activation was increased (*z*‐score = 2.11, *p* = 0.006). Similarity analysis revealed overlapping molecular responses between ageing and disease.

**Main Limitations:**

Small sample size and inclusion of only cheek teeth limit generalisability. Proteomic findings require functional validation.

**Conclusions:**

Ageing and disease induce distinct yet overlapping proteomic alterations in equine dental tissues, primarily affecting ECM homeostasis. A subset of consistently altered proteins may serve as targets for regenerative strategies, with implications for improving equine dental health and welfare.

## 1 INTRODUCTION

Dental disease is an important equine welfare issue as it can significantly affect mastication, occlusal function and comfort of an animal. Its prevalence increases with ageing significantly impacting the welfare of older horses. A UK survey reported that 95.4% of horses examined over 15 years old presented dental abnormalities.^1^ Dental disorders associated with ageing include periodontal disease,^2^, ^3^ diastemata,^4^ pulpitis,^5^ and more recently equine odontoclastic tooth resorption and hypercementosis (EOTRH),^6^ a dental disorder of aged horses, commonly occurring in incisors.^7^

Equine cheek teeth are hypsodont, adapted for continuous eruption to accommodate a high‐fibre diet, which is critical for efficient mastication. This unique dental morphology results in lifelong mechanical stress and cellular turnover, which may contribute to their susceptibility to cumulative age‐related changes. Dental tissue results from the deposition of inorganic crystals of calcium hydroxyapatite on collagenous matrix.^8^ The enamel has a high mineral content (96%–98%) and is acellular impacting its ability for repair.^9^ Dentin on the other hand, is continuously deposited by pulp odontoblasts and consists of a lower mineral content (70%) as well as organic fibre and mucopolysaccharides.^9^ The cementum has an even higher organic content and widespread collagen fibre networks which give it more flexibility. Peripheral cementum is also vascularised, innervated and cellular, with cementoblasts and cementocytes residing in the periodontium/at the periphery of the enamel layer and dentin root.^8^ The dental pulp is a soft connective tissue located at the centre of the tooth.^10^, ^11^ It plays a vital role in dentin formation through the activity of odontoblasts, as well as in maintaining tooth vitality and responding to injury or infection.^12^, ^13^ In equine teeth, the pulp is extensive and complex, with multiple pulp horns in each cheek tooth, and it remains active throughout life due to the continuous eruption and wear of hypsodont teeth.^9^, ^11^

Protein composition is an important determinant of tissue homeostasis and remodelling, and progression of disease. As such, characterising the dental tissue proteome in horses and identifying age‐related modifications in protein composition is important to understand the molecular pathways leading to dental disease in ageing horses. Indeed, proteomic analysis of dental tissue in other species has revealed a complex proteome with immune‐related and inflammation‐modulating proteins identified in addition to major proteins of the extracellular matrix (ECM) such as amelogenin, dentin sialophosphoprotein and bone sialoprotein.^14^, ^15^, ^16^ Additionally, proteomics changes of ECM proteins in teeth from young and old humans have been investigated in humans.^17^

In ageing connective tissues, including those in the oral cavity, cumulative oxidative stress,^18^ collagen crosslinking^19^ and altered matrix turnover^20^, ^21^ are frequently observed, all of which may influence the dentoalveolar proteome. Equine teeth also experience age‐related morphological changes, including an increase in the thickness of the dentin layer and a reduction in the size of the pulp chamber while the density of pulp fibroblasts increases.^22^ However, the proteome of equine teeth and its modification with ageing have not been investigated. Our previous cementum proteomic analysis of horses with EOTRH revealed distinct protein expression patterns within the incisors of diseased teeth.^23^ Here, we analysed the proteome of equine cheek teeth across three groups: mature adult, old and diseased samples. Cheek teeth were chosen for their relevance to mastication and their high susceptibility to age‐related wear and pathology.^8^ This study enabled the characterisation of age‐associated proteomic shifts in healthy and diseased equine dental tissues, providing new insight into the molecular pathways contributing to dental ageing and disease.

## 2 METHODS

### 2.1 Collection of equine cheek teeth

Equine cheek teeth (specifically Triadan 06s and 09s) were collected from cadaver heads of mixed breeds sourced from an abattoir or from clinical cases requiring surgical extraction which was undertaken orally. All teeth used in the study were removed intact; any teeth that fractured during the extraction process were excluded from analysis. Samples were aged using passports and confirmed via dental examinations.

Teeth were allocated to three groups (*n* = 5/group) to separate age‐associated protein changes from disease‐associated changes. Mature Adults represented horses that had reached full dental maturity. Old group represented the period of highest susceptibility to dental pathology, which increases dramatically after 15 years. This group served as an age‐matched control for the diseased group.

Healthy teeth (mature and old) were included only if they showed no visible occlusal pathology, including open pulp horns or infundibular caries, and exhibited ‘normal’ age‐related wear without cupping out. The diseased group was diagnosed with apical infection via oroscopic examination and radiography. The teeth had occlusal abnormalities or associated clinical signs resulting in the need for radiography. Inclusion required at least two of the following radiographic signs: bony sclerosis, blunting of root apices, or apical halos. Clinical cases required extraction and were therefore all classified as severely diseased. A convenience sample number was used due to financial constraints. All tissues were stored immediately at −80°C.

### 2.2 Isolation of dental tissues

Dental tissues were isolated in a sequential process. Briefly, adherent periodontal ligament was removed using a sterile scalpel. Pulp was extracted using sterile dental probes and endodontic files. Both soft tissues were immediately stored at –80°C. Teeth were sectioned into three segments: crown, reserve crown and root. The segments were separated by cutting horizontally using a Precision Diamond Wire Saw (WELL, Le Locle, Switzerland) under constant water cooling. The crown was further cut into 1–3 mm thick sections. To distinguish the individual tissues, the cross sections were visualised under Quantitative Light‐induced Fluorescence imaging.^23^ Any remaining pulp tissue was removed, and hard tissues (dentin and (peripheral) cementum) were cut based on acquired images.

### 2.3 Protein extraction

Protein extraction from hard tissues (cementum, dentin, enamel) was performed using a sequential protocol with ethylenediaminetetraacetic acid (EDTA) (Fisher Scientific) and guanidine hydrochloride (GnHCl) (ThermoFisher). Briefly, cementum powder was first incubated in phosphate‐buffered saline (ThermoFisher) for 5 min with constant agitation, followed by centrifugation at 10,000 × g, and the supernatant was discarded. The pellet was then re‐suspended in 0.5 M EDTA (pH 8.0) containing cOmplete™ EDTA‐free Protease Inhibitor Cocktail (Merck) at 4°C under constant motion. After 2 days, the sample was centrifuged, and the supernatant was collected. Fresh EDTA was added, and the sample underwent sonication for ten 30‐s cycles using a Bioruptor Sonication Device (Diagenode). After a further 3 days, the second EDTA extract was collected. Finally, the remaining pellet was incubated with a GnHCl extraction buffer containing 4 M GnHCl (ThermoFisher), 65 mM DTT (ThermoFisher) and 50 mM sodium acetate (Invitrogen) at 4°C with agitation for 3 days. All protein extracts were pooled for each individual sample for downstream analysis. Dental pulp was removed from teeth using endodontic files and homogenised. Proteins were extracted from 50 mg of dental pulp using the same 4 M GnHCl extraction buffer as above.

### 2.4 Liquid chromatography tandem mass spectrometry

Samples were desalted using C18 ZipTips (MilliporeSigma) according to the manufacturer's instructions, then centrifuged and transferred to total recovery vials (Waters) for high‐resolution liquid chromatography–tandem mass spectrometry (LC–MS/MS) analysis. Approximately 300 ng of each sample was loaded onto the trapping column (PepMap100, C18, 300 *μ*m × 3.5 mm; ThermoFisher Scientific) using partial loop injection for 7 min at a flow rate of 12 *μ*L/min with 0.1% (v/v) formic acid. Peptides were separated on the analytical column (Easy‐Spray C18, 75 *μ*m × 500 mm; ThermoFisher) using a gradient from 96.2% solvent A (0.1% formic acid in water; Merck) and 3.8% solvent B (79.95% acetonitrile, 19.95% water, 0.1% formic acid; Merck) to 50% A/50% B over 90 min at a flow rate of 0.3 nL/min, followed by 30‐min blanks between samples.

Data acquisition employed a data‐dependent program with a full‐scan MS resolution of 60,000 in the Orbitrap (ThermoFisher) (automatic gain control [AGC] target: 3 × 10^6^ ions; maximum fill time: 100 ms). The 16 most intense peaks from each full scan were selected for higher energy collisional dissociation MS/MS at 30,000 resolutions (AGC target: 1 × 10^5^ ions; maximum fill time: 45 ms), with an isolation window of 2 m/z and normalised collision energy of 30%. Singly charged ions and ions with charge states ≥+6 were excluded. To prevent repeated selection of the same peptides, a dynamic exclusion window of 60 s was applied. All samples were analysed in a randomised order.

### 2.5 Label‐free quantification

Peptide and protein identification was performed using an in‐house Mascot server. Searches were conducted against the UniHorse database with the following parameters: peptide mass tolerance of 10 ppm, fragment mass tolerance of 0.01 Da, trypsin as the enzyme with up to one missed cleavage, fixed modification of carbamidomethylation (cysteine), and variable modification of oxidation (methionine). Protein quantification was carried out using Progenesis QI software v4 (Waters), considering only unique peptides. All four datasets have been uploaded to the PRIDE submission database under the following identifiers: Enamel (PXD065888), Dentin (PXD065894), Cementum (PXD066267), and Pulp (PXD065888).

### 2.6 Data analysis

Proteins were filtered to exclude those with excessive missing values; specifically, only proteins quantified in at least three biological replicates per group were retained for further analysis. Data was median normalised and pairwise differential expression analysis was performed within each tissue type using NormalyzerDE,^24^ comparing the following groups: mature adult vs. old, and old vs. diseased. Statistical significance was assessed using empirical Bayes moderated *t*‐tests. Proteins were considered differentially abundant if they met both a fold change threshold of log_2_(FC) ≥1 or ≤−1 and a *p*‐value ≤0.05. All visualisations were generated using the ggplot2 package (version 3.4.3) in R. The clusterProfiler R package (version 4.6.2) was used for gene ontology (GO) and pathway over‐representation analysis, allowing the identification of enriched biological processes, molecular functions and cellular components associated with protein abundance changes. Ingenuity pathway analysis (IPA) (QIAGEN Inc.)^25^ was employed to provide complementary insights, including upstream regulator prediction, canonical pathway mapping and network‐level interpretation of the data. Differential abundance data was input into IPA and the data was cross referenced against the ‘user dataset’; all proteins identified in the experiment.

### 2.7 Shiny app

A custom Shiny application was developed in R (version 4.2.2) using the shiny R package (version 1.7.4.1). Within the app, protein expression data and differential abundance analysis outputs from the four dental tissues were integrated. Pairwise comparisons were organised to separately evaluate ageing (old vs. mature) and disease (old diseased vs. old healthy). The application includes modules for generating interactive volcano plots, visualising overlap of differentially abundant proteins across tissues and exporting datasets. The app was deployed online at https://andersjensen14.shinyapps.io/ageing_teeth_shiny/, and the source code is publicly available at https://github.com/AJensen14/Teeth_Shiny_App.

## 3 RESULTS

### 3.1 Mass spectrometry proteomics revealed tissue‐specific protein signatures

Cheek teeth taken from mature adult, old and diseased horses were used for individual tooth tissue isolation and proteomic analysis. Mature samples had a mean age of 7.0 years old (SD ±1.22) and were significantly younger than both old (mean; 19.8 years old, SD ±2.59) and diseased samples (mean; 19.4 years old, SD ±3.05). There was no significant difference in age between the old and diseased group (Figure 1A). Details of age, location tooth extracted from and tooth type are in Table S1. Proteomic analysis identified a mean of 570 proteins in cementum (SD = ±31.1), 517 in dentin (SD = ±5.28), 254 in enamel (SD = ±5.49) and 1636 in pulp (SD = ±8.55) (Figure 1B).

**FIGURE 1:**
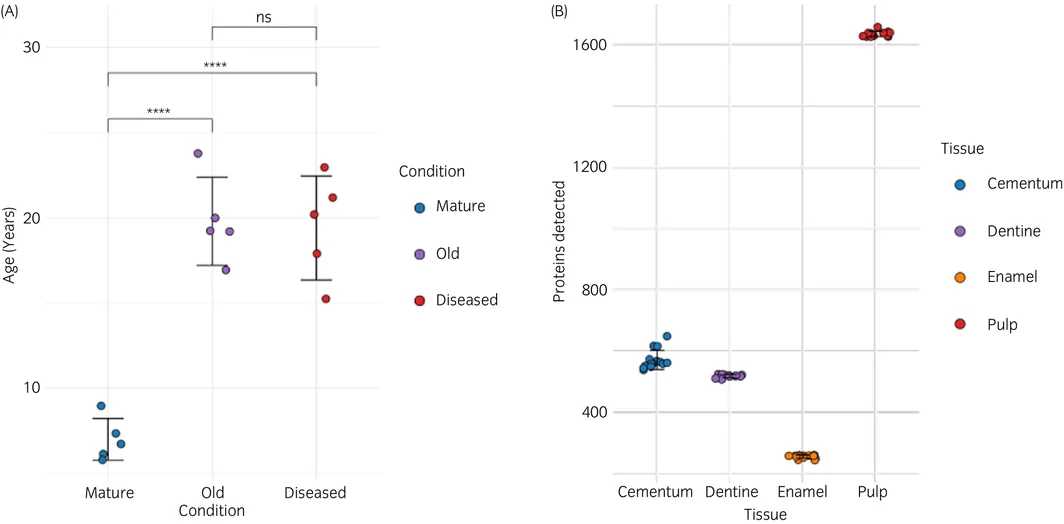
Overview of equine cheek teeth proteomics and sample characteristic. (A) Age distribution of samples across three experimental groups. (B) Number of proteins detected for each sample in each tissue (cementum, dentin, enamel, pulp) used in the study. Each point represents a sample, and the error bars indicate SD. Significant differences between groups are indicated by (****p < 0.0001, ***p < 0.001, **p < 0.01, *p < 0.05). Figures were produced using the ggplot2 R package (version 3.5.2).

Following protein identification and quantification, all proteins identified in at least one biological replicate across all three sample groups were compared across tissues to characterise dental tissue‐specific protein profiles. The overlap of detected proteins is illustrated in Figure 2. Among the 1950 proteins detected, more than half (~58%) were unique to dental pulp, while approximately 6% were commonly detected across all four tissues. Unique enamel proteins included collagen type XVII (COL17A1) and kallikrein‐4 (KLK4). Proteins specific to dentin included tissue inhibitor of metalloproteinases 3 (TIMP3) and procollagen‐lysine, 2‐oxoglutarate 5‐dioxygenase 2 (PLOD2). Cementum‐associated proteins included matrix metalloproteinase 9 (MMP9) and insulin‐like growth factor‐binding protein 3 (IGFBP3). The dental pulp proteome was markedly larger than that of other dental tissues and uniquely featured numerous cellular proteins including stathmin‐2 (STMN2), retinoblastoma‐binding protein 4 (RBBP4), integrin alpha‐M (ITGAM), and isocitrate dehydrogenase (NADP), mitochondrial (IDH2).

**FIGURE 2:**
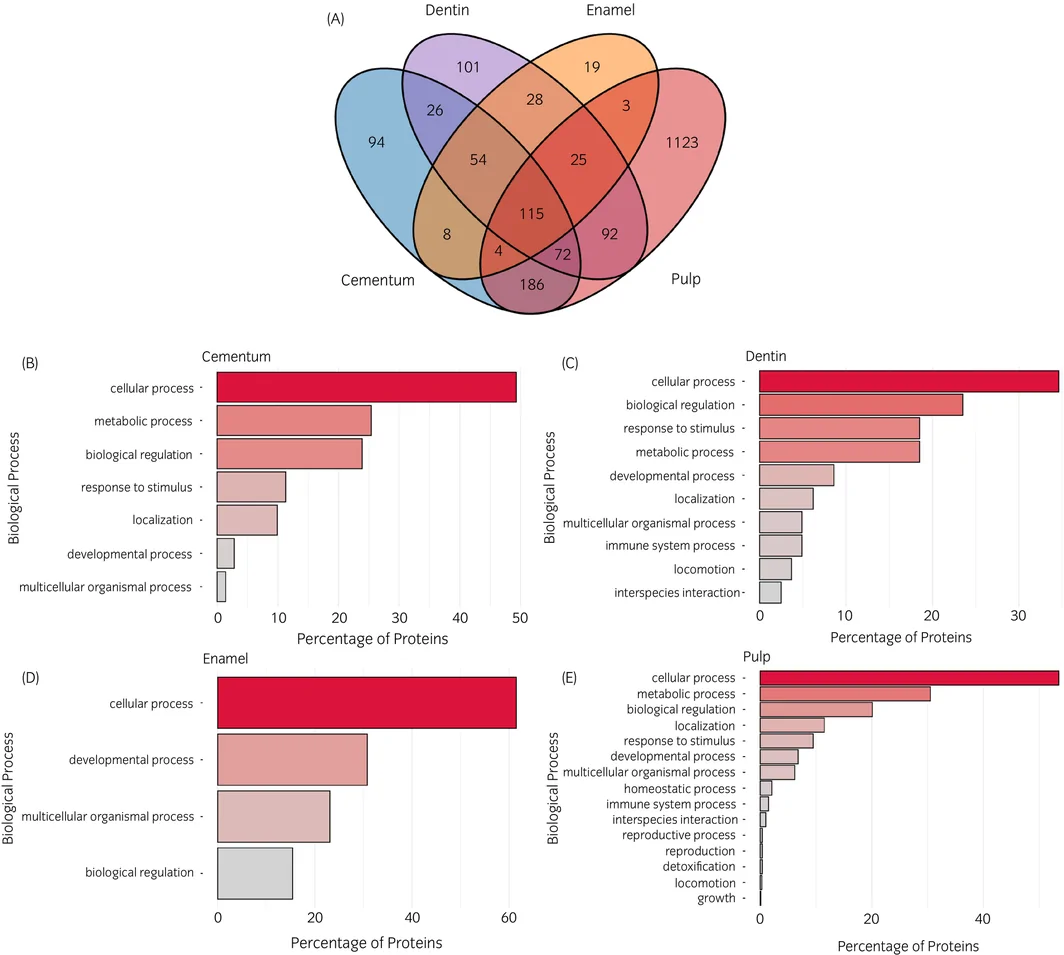
Overlap of proteins detected in different equine dental tissues. (A) Venn diagram showing the overlap of proteins detected in each dental tissue from cheek teeth. Colours represent different tissues, and the numbers show the number of proteins in each category. Figure produced using the ggvenn R package (version 0.1.10). (B) Biological processes enriched in tissue‐specific proteins in dental tissues. Barplots for each tissue are for (B) cementum, (C) dentin, (D) enamel and (E) pulp. Unique proteins to each dental tissue were identified and analysed using PANTHER (protein analysis through evolutionary relationships) classification system to determine enrichment in biological processes based on GO annotations. Figures produced using the ggplot2 R package (version 3.5.2).

Proteins exclusively detected in a single dental tissue were further characterised using PANTHER.^26^ The biological processes associated with these protein lists were determined (Figure 2B–E). Due to the large number of uniquely identified proteins in dental pulp, we further investigated their functional roles using clusterProfiler R (Figure S1).

### 3.2 Cluster analysis revealed age and disease differences

To explore the variability among mature adult, old and diseased samples, we applied unsupervised clustering techniques to each dental tissue type. Principal component analysis was conducted on normalised protein intensity data, restricted to complete observations. The first two principal components explained 38.4% (cementum), 40.5% (dentin), 34.7% (enamel) and 50.8% (pulp) of the total variance (Figure S2).

### 3.3 Differential abundance analysis revealed age and disease differences

Protein quantities were compared between groups within each dental tissue to identify ageing‐ and disease‐related changes. Proteins were considered significantly different if they showed a log_2_ fold change >±1 and *p* < 0.05. Across all tissues, ageing was associated with 173 increased and 156 reduced proteins, while disease was linked to 160 increases and 219 reductions. Figure 3 summarises these results.

**FIGURE 3:**
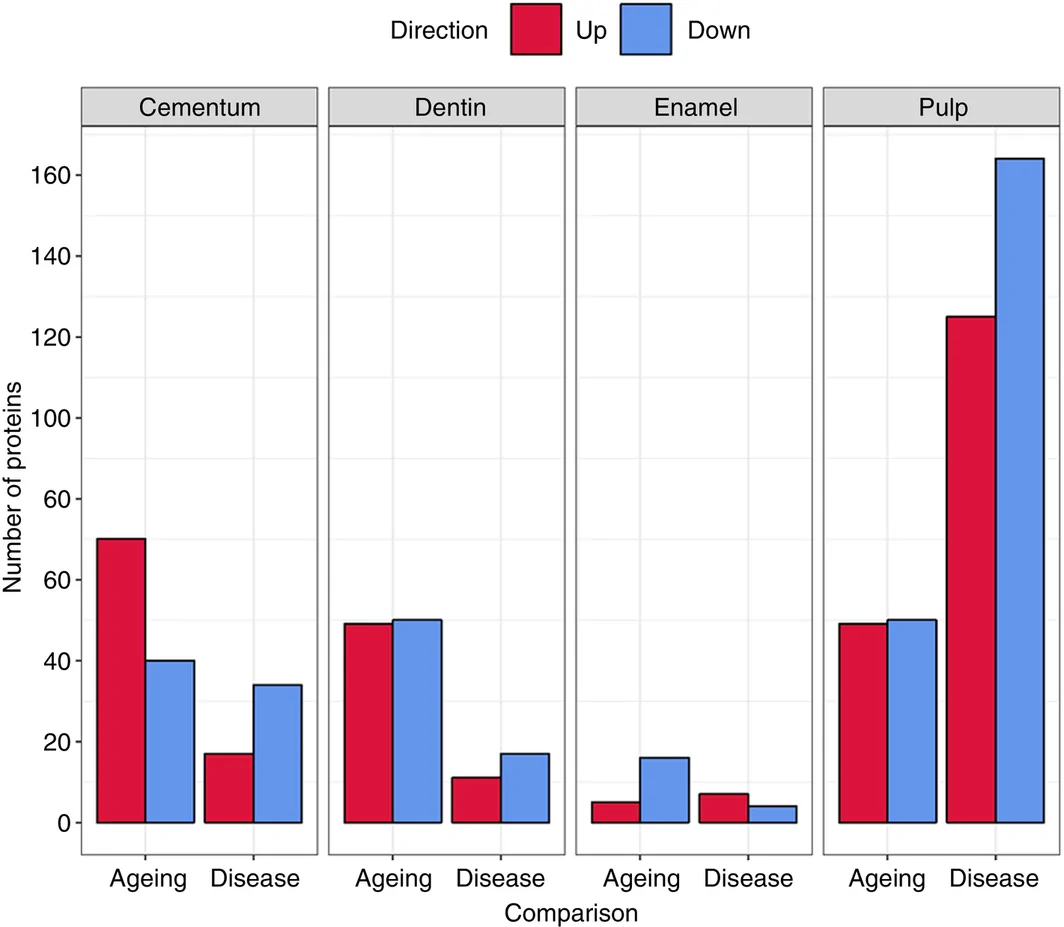
Summary of differential abundance analysis. Bar charts display the number of significantly increased and decreased proteins in cementum, dentin, enamel and pulp. Label‐free quantification values were normalised, and differential abundance was assessed using the NormalyzerDE R package (version 1.16.0). Proteins were considered differentially abundant if the log_2_ (fold change) exceeded ±1 and the *p*‐value from pairwise comparisons <0.05. Comparisons were performed for ageing (old vs. mature adult) and disease (diseased vs. old), with up direction indicating higher abundance in old and diseased samples, respectively. Figure was produced using ggplot2 R package (version 3.5.2).

#### 3.3.1 Cementum

To observe differential abundance across each dental tissue, volcano plots were used to visualise protein changes in ageing and disease. In cementum there were 110 proteins significantly different in ageing (Figure 4A) whereas 51 proteins significantly changed in disease (Figure 4B) (*p* < 0.05, logFC >1). In cementum samples, the metalloproteinases MMP‐2 and MMP‐13 were both increased in older teeth along with other markers of ageing including calreticulin (CALR) and alkaline phosphatase (ALPL). Cellular proteins such as S‐adenosylhomocysteine hydrolase (AHCY), chaperonin containing TCP1 subunit theta (CCT) and family with sequence similarity 171 member B (FAM171B) were more abundant in mature adult samples, as were ECM proteins such as Inter‐alpha‐trypsin inhibitor heavy chain H2 (ITIH2) and Carbonic anhydrase 2 (CA2) (Figure 4A). Collagen type XV (COL15A1) was less abundant in diseased samples compared with healthy older samples (Figure 4B).

**FIGURE 4:**
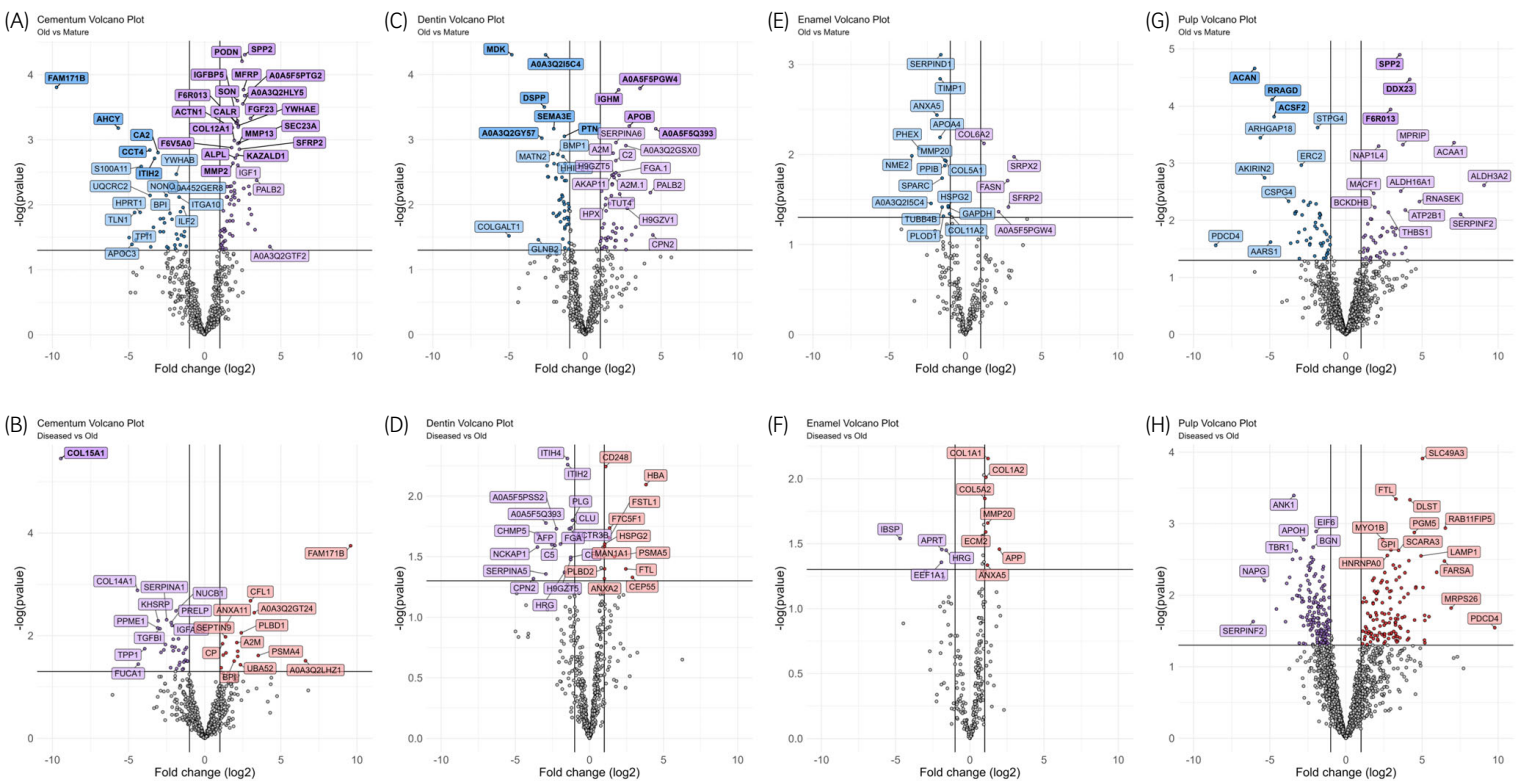
Differential abundance in cheek teeth tissues. Differential abundance analysis of cementum, dentin, enamel and pulp. (A, C, E, G) Volcano plots comparing protein abundance across ageing for cementum, dentin, enamel and pulp and (B, D, F, H) for disease across cementum, dentin, enamel and pulp. Differential abundance analysis was carried out using NormalyzerDE R package (version 1.16.0) for cementum proteomics data. The *x*‐axis represents the log_2_ fold change (FC) in protein abundance, and the *y*‐axis shows the –log10(*p*‐value) indicating statistical significance. Points represent individual proteins: Coloured points indicate significantly upregulated or downregulated proteins based on *p*‐value <0.05 and log_2_ FC >1 thresholds. Grey points are proteins not significantly changed. Vertical dashed lines at ±1 mark the fold change cutoffs, and the horizontal dashed line indicates the significance threshold (*p* = 0.05). Labels with bolded text and higher opacity are significant after *p*‐value adjustment. Figures were produced using ggplot2 R package (version 3.5.2).

#### 3.3.2 Dentin

For dentin samples there were 99 and 28 proteins differentially abundant with age and disease respectively (*p* < 0.05, logFC >1). The immunoglobulin heavy chain protein (IGHM) was increased in old horses compared with mature adults. Midkine (MDK) and pleiotrophin (PTN) were two growth factors with higher abundance in dentin from mature adults. Dentin sialophosphoprotein (DSPP) was reduced in older samples (Figure 4C). In dentin from diseased cheek teeth, several proteins were reduced when compared with age‐matched healthy teeth including Serpin family A member 2 (SERPINA2), Clusterin (CLU), Plasminogen (PLG), Alpha‐fetoprotein (AFP) and Inter‐alpha‐trypsin inhibitor heavy chain H4 (ITIH4). In contrast, Annexin A2 (ANXA2), Haemoglobin subunit alpha (HBA), Proteasome subunit alpha type‐5 (PSMA5) and Mannosidase alpha class 1A member 1 (MAN1A1) were increased in disease (Figure 4D).

#### 3.3.3 Enamel

Enamel had the smallest number of proteins identified and demonstrated the lowest number of significantly different proteins (Figure 4E,F). There were 16 proteins reduced in older samples compared to mature adult and five proteins in higher abundance in old samples (*p* < 0.05, logFC >1). In disease there were 11 differentially abundant proteins. The metalloproteinase enamelysin (MMP20) was reduced with ageing, but more abundant in the diseased old samples compared to age‐matched healthy samples. In ageing TIMP1, serpin family D member 1 (SERPIND1) and annexin A5 (ANXA5) were more abundant in mature adult samples, whereas sushi repeat‐containing protein X‐linked 2 (SRPX2), collagen type VI alpha 2 chain (COL6A2), fatty acid synthase (FASN) and secreted frizzled‐related protein 2 (SFRP2) were elevated in older samples (Figure 4E). In the comparison of old versus diseased enamel, integrin‐binding sialoprotein (IBSP), adenine phosphoribosyltransferase (APRT) and histidine‐rich glycoprotein (HRG) were reduced in disease, while collagen type I alpha 1 chain (COL1A1), amyloid precursor protein (APP), collagen type V alpha 2 chain (COL5A2) and collagen type I alpha 2 chain (COL1A2) were elevated.

#### 3.3.4 Pulp

Dental pulp had the highest number of significantly different proteins with 99 and 289 proteins impacted by ageing and disease respectively (*p* < 0.05, logFC >1) (Figure 4G,H). Secreted phosphoprotein 2 (SPP2), dead‐box helicase 23 (DDX23), and matrix‐gla protein (F6R013) were more abundant in ageing (Figure 4G). Aggrecan (ACAN), ras‐related GTP‐binding protein D (RRAGD), and acyl‐coA synthetase family member 2 (ACSF2) were reduced with ageing (Figure 4G). In disease, we found solute carrier family 49 member 3 (SLC49A3), ferritin light chain (FTL) and dihydrolipoamide S‐succinyltransferase (DLST) were more abundant. In contrast, ankyrin 1 (ANK1), apolipoprotein H (APOH) and eukaryotic translation initiation factor 6 (EIF6) were reduced in diseased pulp (Figure 4H).

After differential‐abundance analysis, we highlighted the top 30 proteins per tissue (raw *p*‐values). Cementum showed three proteins altered in both comparisons, dentin six, enamel nine and dental pulp only one (Tables [Link], [Link], [Link], [Link]).

### 3.4 Cross tissue comparisons of differential abundance

To determine proteins with potential roles in ageing or disease across dental tissues we explored patterns of overlap in differentially abundant proteins (*p* < 0.05, logF >1) between tissues for each comparison (age and disease). Figure 5A shows the overlap of increased proteins in ageing across tissues. Five proteins were increased in enamel and four of those proteins were also increased in different dental tissues, including COL6A2, SRPX2, SFRP2 and uncharacterised protein A0A5F5PGW4. In both dentin and enamel, MMP‐20, ANXA5 and COL5A1 were reduced in older samples compared to mature adults. Versican (VCAN) and follistatin‐like 1 (FSTL1) were reduced as a result of ageing in dental pulp and dentin (Figure 5B).

**FIGURE 5:**
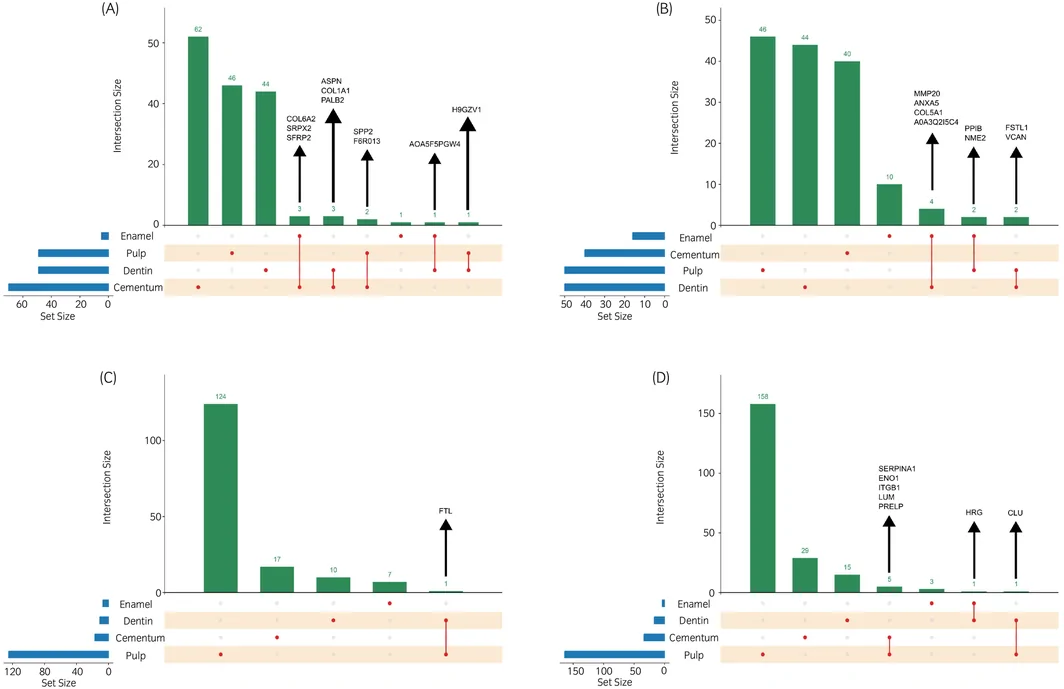
UpSet plot showing proteins associated with ageing (A, B) and disease (C, D) across different dental tissues. A. Proteins with an increased abundance in old samples. (B) Proteins with an increased abundance in mature samples. (C) Proteins with an increased abundance in diseased samples. (D) Proteins with an increased abundance in age‐matched healthy samples. The plot displays intersections of proteins that are significantly altered in old versus mature adult comparisons across cementum, dentin, enamel and pulp (*p* < 0.05 and |logFC| >1). Each horizontal bar represents the total number of significant proteins identified per tissue. The connected vertical bars indicate the number of shared proteins among the respective tissues. Figures were made using the UpSetR package (version 1.4.0).

Patterns of overlap in differentially abundant proteins (*p* < 0.05, logFC >1) in disease between tissues were further explored. Figure 5C shows the pattern of increased protein abundance in diseased samples compared with old‐healthy samples. Ferritin light chain (FTL) was increased in both pulp and dentin of diseased teeth. Cementum and pulp shared the greatest overlap in reduced proteins, with serpin family A member 1 (SERPINA1), alpha‐enolase (ENO1), integrin beta‐1 (ITGB1), lumican (LUM) and prolargin (PRELP) all reduced in disease (Figure 5D).

### 3.5 Pathway analysis revealed commonly enriched pathways across dental tissues

We used over‐representation analysis to interpret functional shifts in differentially abundant proteins across the four dental tissues. Enriched pathways reflected the molecular patterns observed: ageing‐associated increases were largely linked to ECM organisation in all tissues. Dental pulp showed a broader representation of cellular processes than the hard tissues (enamel, dentin, cementum) (Figure 6).

**FIGURE 6:**
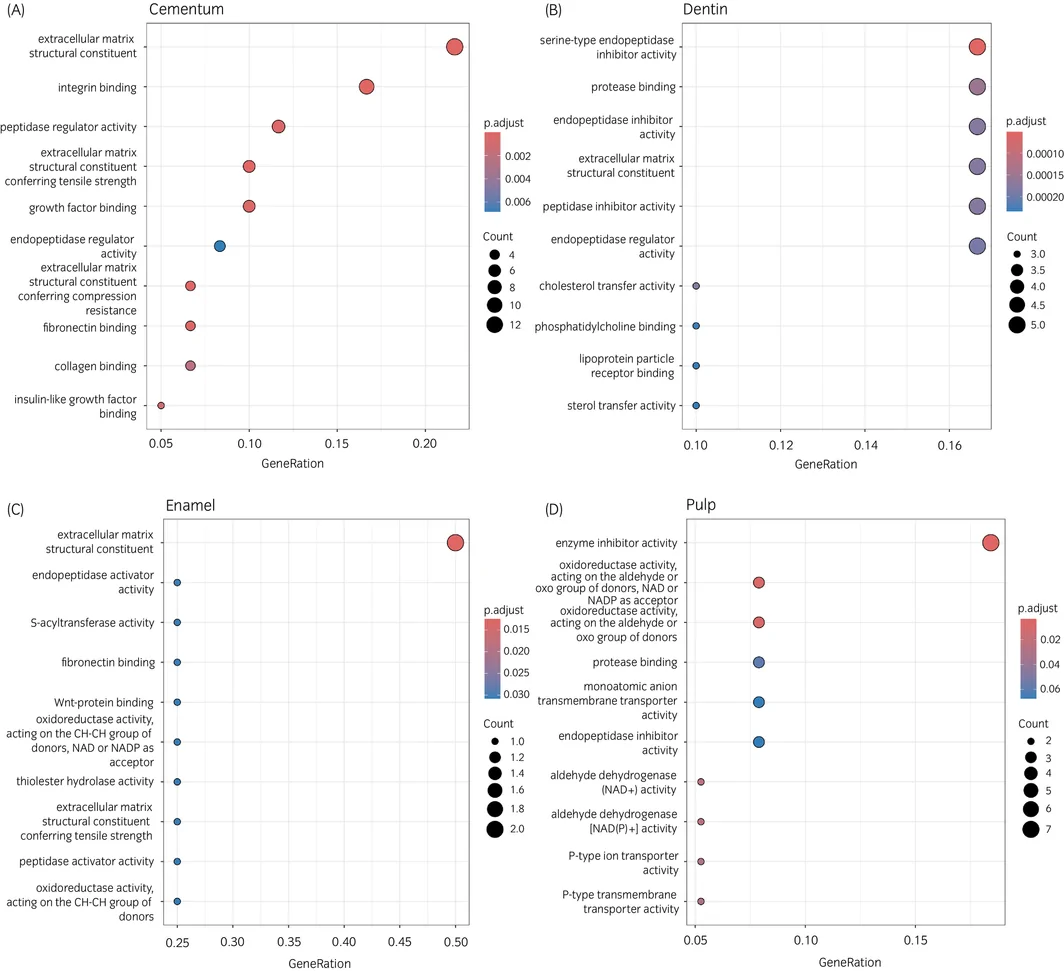
Over‐representation analysis of proteins increased in ageing across dental tissues. The top 10 molecular function pathways are shown for (A) cementum, (B) dentin, (C) enamel and (D) pulp. Proteins showing significantly increased abundance in old samples compared to mature adult samples were identified through differential abundance analysis of proteomics data using the NormalyzerDE R package (version 1.16.0). For each dental tissue type, increased proteins were analysed separately. Over‐representation analysis was performed using the ClusterProfiler R package (version 4.6.2), focusing on Gene Ontology (GO) molecular function terms. In the dotplots, colour represents the adjusted *p*‐value (Benjamini‐Hochberg correction). Dot size corresponds to the number of up‐regulated proteins associated with each GO term and the gene ratio on the *x*‐axis denotes the proportion of up‐regulated genes mapped to a given GO term relative to the total number of up‐regulated proteins used in the analysis.

Conversely, molecular functions reduced in ageing showed a prominent involvement of various enzyme activities, such as endopeptidase, ligase and lipase activity for cementum, enamel and pulp (Figure 7). Furthermore, functions related to ECM alterations were observed as downregulated across all tissues, except for cementum (Figure 7A).

**FIGURE 7:**
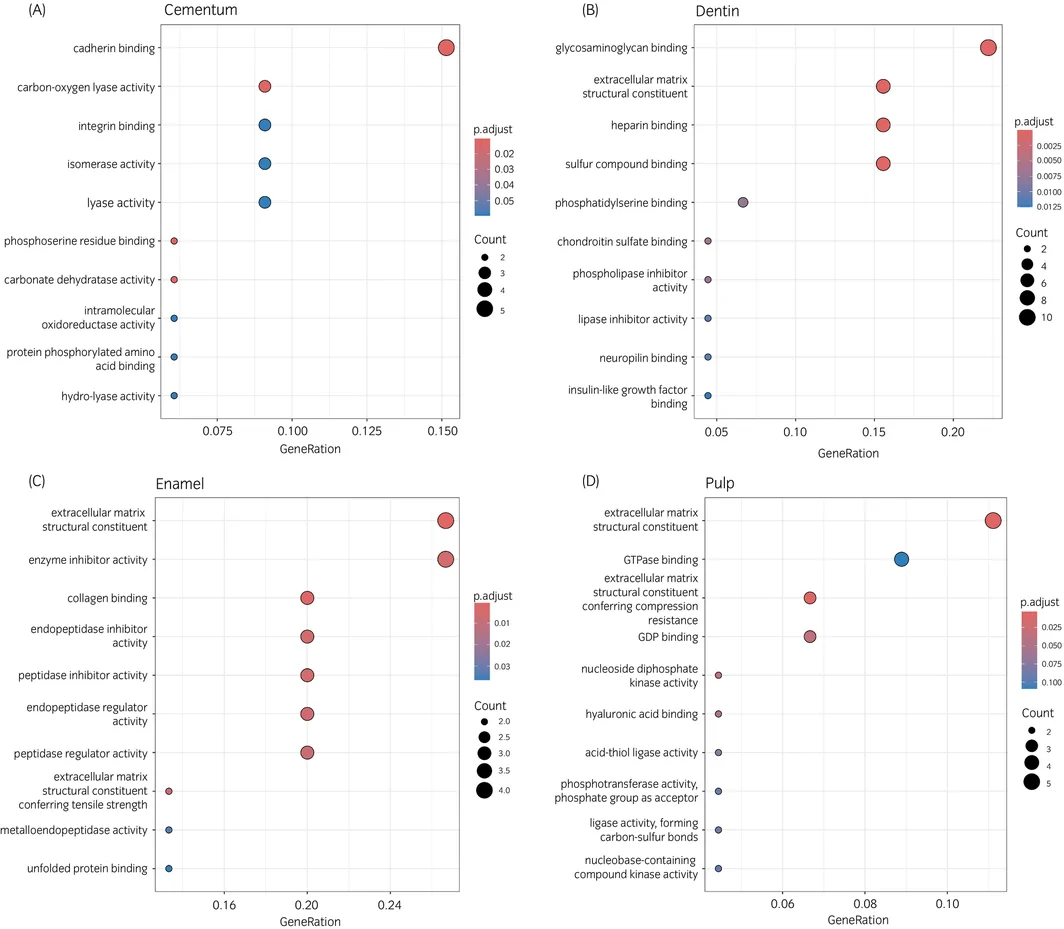
Over‐representation analysis of proteins reduced in ageing across dental tissues. The top 10 molecular function pathways are shown for (A) cementum, (B) dentin, (C) enamel and (D) dental pulp. Proteins showing significantly decreased abundance in aged samples compared to mature adult samples were identified through differential abundance analysis of proteomics data using the NormalyzerDE R package (version 1.16.0). For each dental tissue type, reduced proteins were analysed separately. Over‐representation analysis was performed using the ClusterProfiler R package (version 4.6.2), focusing on GO molecular function terms. In the dotplots, colour represents the adjusted *p*‐value (Benjamini‐Hochberg correction). Dot size corresponds to the number of up‐regulated genes associated with each GO term and the gene ratio on the *x*‐axis denotes the proportion of up‐regulated proteins mapped to a given GO term relative to the total number of up‐regulated proteins used in the analysis.

In contrast to the age‐related changes, the comparison of diseased against healthy age‐matched cheekteeth revealed fewer overall significant alterations in molecular function using proteins which increased in abundance with disease (Figure 8). Only enamel and dental pulp exhibited significantly enriched functions (adjusted *p*‐value <0.05). In enamel, these alterations primarily focused on changes within the ECM (Figure 8C), whereas in dental pulp, an increase in cellular proteins, such as cadherin binding (*p* < 0.01) and guanyl ribonucleotide binding (*p* < 0.05) was observed in diseased teeth (Figure 8D).

**FIGURE 8:**
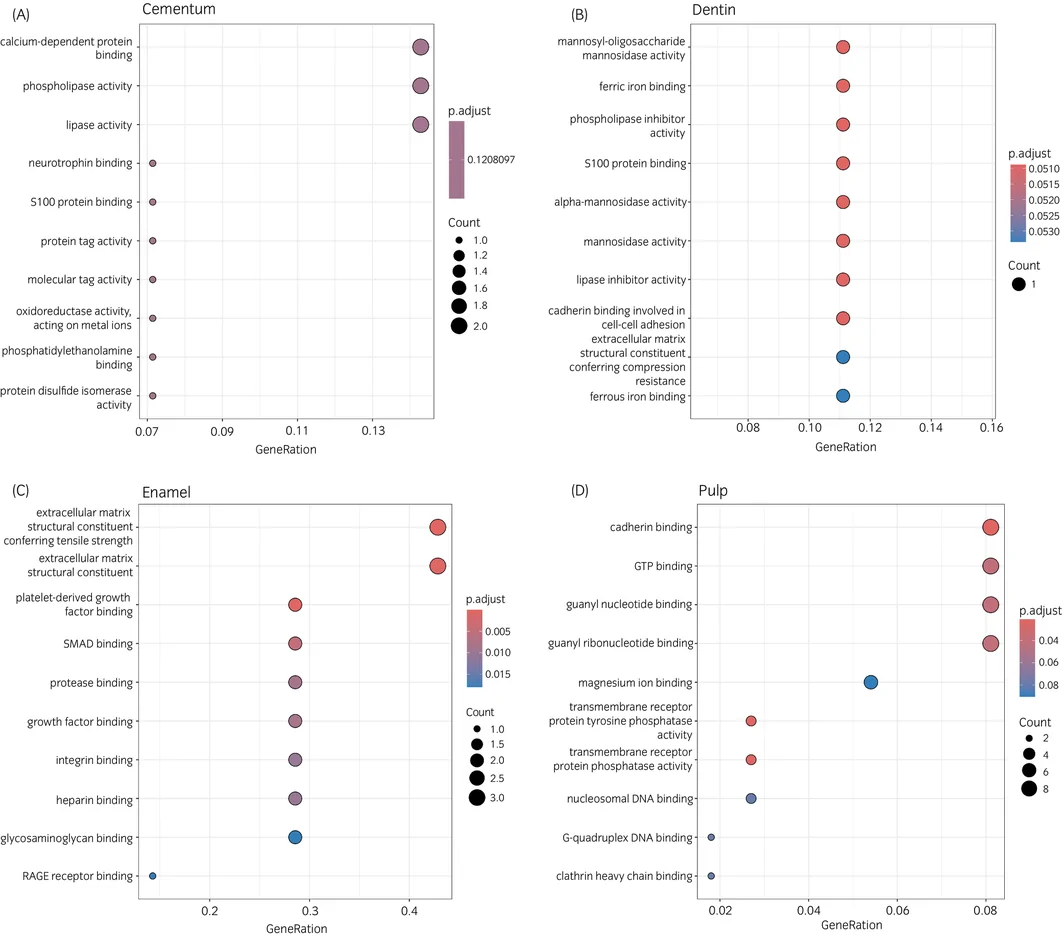
Over‐representation analysis of proteins increased in disease across dental tissues. The top 10 molecular function pathways are shown for (A) cementum, (B) dentin, (C) enamel and (D) dental pulp. Proteins showing significantly increased abundance in diseased tissues compared to healthy old samples were identified through differential abundance analysis of proteomics data using the NormalyzerDE R package (version 1.16.0). For each dental tissue type, up‐regulated proteins were analysed separately. Over‐representation analysis was performed using the ClusterProfiler R package (version 4.6.2), focusing on GO molecular function terms. In the dotplots, colour represents the adjusted *p*‐value (Benjamini‐Hochberg correction). Dot size corresponds to the number of up‐regulated genes associated with each GO term and the gene ratio on the *x*‐axis denotes the proportion of up‐regulated proteins mapped to a given GO term relative to the total number of up‐regulated proteins used in the analysis.

Finally, we described pathway changes for dental tissues in which proteins were reduced in abundance in disease (Figure 9). In the cementum of diseased teeth, the highlighted pathways were involved in ECM maintenance through interactions with various molecules, such as integrins, collagens, laminins, and fibronectin (Figure 9A). In dentin (Figure 9B) and pulp (Figure 9C), there was an overrepresentation of proteins involved in the regulation of peptidase regulation in the age‐matched healthy cheek teeth.

**FIGURE 9:**
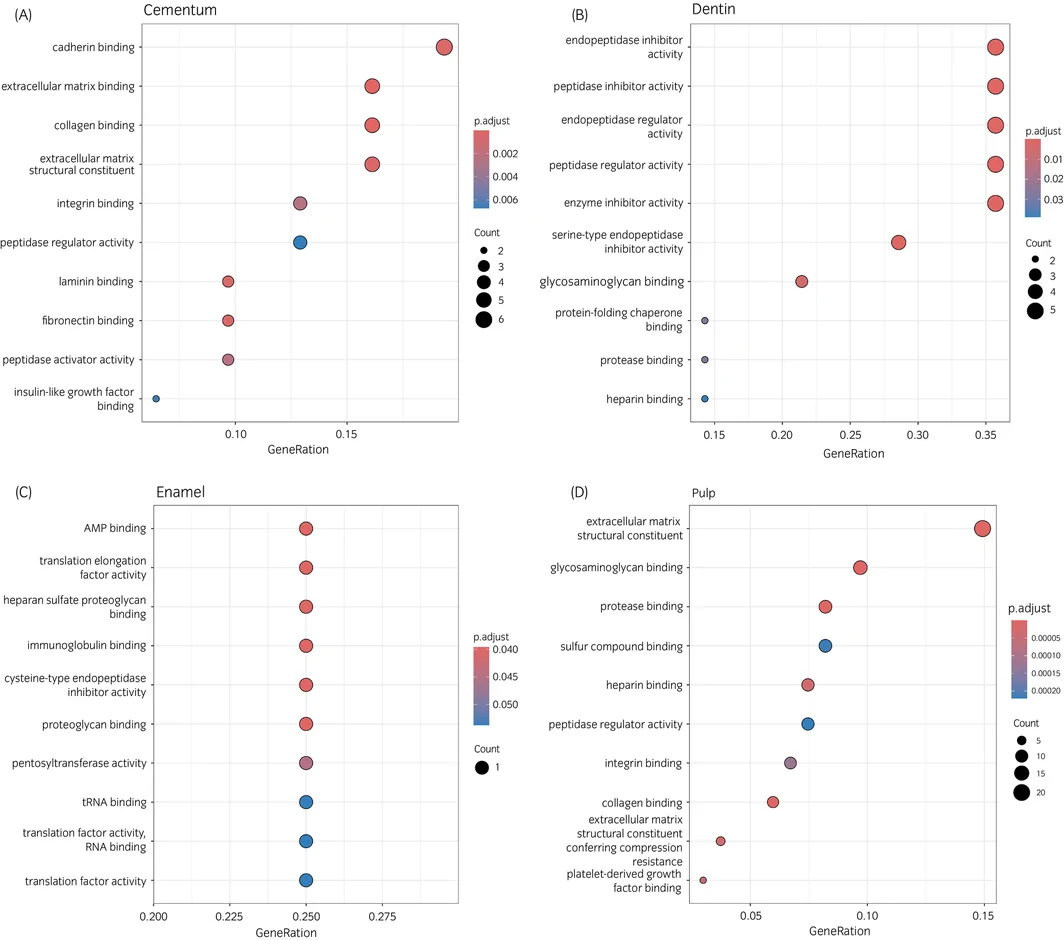
Over‐representation analysis of proteins reduced in disease across dental tissues. The top 10 molecular function pathways are shown for (A) cementum, (B) dentin, (C) enamel and (D) dental pulp. Proteins showing significantly decreased abundance in diseased tissues compared to age‐matched healthy samples were identified through differential abundance analysis of proteomics data using the NormalyzerDE R package (version 1.16.0). For each dental tissue type, up‐regulated proteins were analysed separately. Over‐representation analysis was performed using the ClusterProfiler R package (version 4.6.2), focusing on GO molecular function terms. In the dotplots, colour represents the adjusted *p*‐value (Benjamini‐Hochberg correction). Dot size corresponds to the number of up‐regulated genes associated with each GO term and the gene ratio on the *x*‐axis denotes the proportion of up‐regulated genes mapped to a given GO term relative to the total number of up‐regulated genes used in the analysis.

To further explore the changes observed in diseased dental pulp we utilised IPA as it was the only tissue with enough differentially abundant proteins to permit meaningful interpretation. As shown in Figure 10 several canonical pathways were predicted to be activated in disease. These included retinoic acid receptor activation (*z*‐score = 2.11, *p* = 0.0060), caudal‐type homeobox transcription factors gastrointestinal cancer signalling pathway (*z*‐score = 1.34, *p* = 0.020) and sheddase signalling pathway (*z*‐score = 1.41, *p* = 0.023). Conversely, some canonical pathways were predicted to be inhibited, including regulation of insulin‐like growth factor (IGF) transport and uptake by IGFBPs (*z*‐score = −3.00, *p* = 0.0019), ECM organisation (*z*‐score = −3.05, *p* = 0.032), elastic fibre formation (*z*‐score = −2.82, *p* = 0.0033), integrin cell surface interactions (*z*‐score = −2.49, *p* = 0.00065) and post‐translational protein phosphorylation (*z*‐score = −2.50, *p* = 0.0087). Pathways related to ECM components, such as collagen biosynthesis and modifying enzymes (*z*‐score = −2.33, *p* = 0.0029) and syndecan interactions (*z*‐score = −2.45, *p* = 0.017), also showed predicted inhibition in diseased pulp.

**FIGURE 10:**
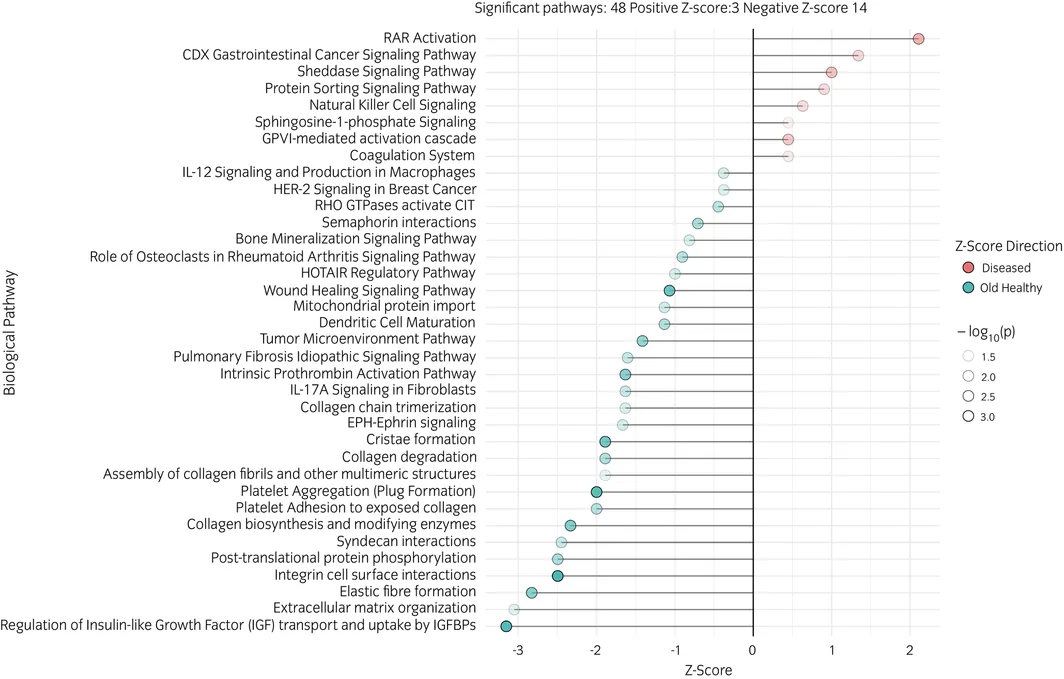
Ingenuity pathway analysis of differentially abundant proteins in diseased pulp. Proteins showing differential abundance between diseased pulp and healthy age‐matched pulp were identified through proteomic analysis. A significance threshold of log_2_ fold change ≥1 and *p*‐value ≤0.05 was applied to define differentially abundant proteins. In the resulting plot, canonical pathways are shown on the *y*‐axis. The *x*‐axis displays the activation *z*‐score, which predicts the activation state of each pathway (positive values indicate activation; negative values indicate inhibition). The opacity of each bar corresponds to the statistical significance of pathway enrichment, represented as –log_10_(*p*‐value), with more opaque bars indicating stronger significance.

### 3.6 Shiny app for interactive visualisation and interpretation of ageing and disease protein changes

A custom Shiny app was built to explore the study's proteomics data, allowing interactive access to protein profiles and differential‐abundance results across the four dental tissues. Ageing (old vs. mature adult) and disease (old diseased vs. old healthy) were analysed separately. The app provides volcano plots, protein‐overlap views, and data‐download options. The full app is available (https://andersjensen14.shinyapps.io/ageing_teeth_shiny/) and source code is available at (https://github.com/AJensen14/Teeth_Shiny_App).

## 4 DISCUSSION

To determine the unique protein signatures in each tissue type, we implemented a label‐free quantitative proteomics approach, using established protocols for mineralised and soft dental tissues.^15^, ^17^, ^27^ Despite dental disease being highly prevalent in horses, in particular with ageing, and the significant impact of dental pathology on horses' welfare, the molecular pathways leading to dental disease are relatively unclear and the proteome of equine cheek teeth has not been previously investigated.^1^, ^28^

Proteomic profiling of equine cheek‐tooth tissues identified 1950 proteins in total: 261 in the acellular, highly mineralised enamel; 523 in dentin; 564 in cementum; and 1641 in the vascularised, highly cellular pulp. As expected, pulp contained the most proteins, reflecting its cellularity and diverse functions. Dentin and cementum showed similar protein counts, consistent with their structural similarities. Protein yield appeared inversely related to mineral content, a pattern also reported in human dental proteomics.^15^, ^29^, ^30^, ^31^

The pulp showed the most complex profile, with 58% (1123) of its proteins unique and only 6% shared across all tissues. Its unique proteins were mainly cellular and linked to repair and angiogenesis, consistent with the pulp's vascularity and its role in sustaining dentin production.^22^ Biological‐process analysis was dominated by metabolic and respiratory functions, and no ECM‐related terms were enriched. Enamel's unique proteins were associated with the extracellular space and collagen, reflecting its acellular nature.^32^ The presence of tissue‐specific proteins supports successful tissue isolation; for example, KLK4, an enamel‐specific serine protease, is essential for enamel matrix processing during maturation.^33^, ^34^ It was exclusively detected in enamel samples, supporting an accurate sample processing procedure. However, the enamel‐specific proteins amelogenin X‐linked (AMELX), ameloblastin (AMNB) and enamelin (ENAM) were identified in enamel and dentin, similar to human dentin studies, possibly due to their presence in the amelodentinal junction.^17^, ^35^ Several proteins were uniquely identified in dentin, notably TIMP3, a regulator of MMP activity, suggesting a tightly controlled environment for ECM remodelling.^36^ This reflects the structural demands of dentin, where matrix turnover is regulated to maintain hardness and prevent pathological degradation. Additionally, dentin‐specific detection of bone morphogenic protein (BMP1)^37^ and a disintegrin and metalloproteinase with thrombospondin motifs 3 (ADAMTS3)^38^ supports ongoing ECM processing. The presence of secreted frizzled‐related protein 1 (SFRP1) and frizzled‐Related protein (FRZB),^39^ both antagonists of Wnt signalling, aligns with previous findings that Wnt modulation influences odontoblast differentiation.^40^ Together, these proteins revealed a profile consistent with tissue‐specific ECM maturation and regulation in the mineralised dentin compartment. Unique proteins identified in cementum also related to the extracellular space and cell matrix interactions, vesicles and interestingly the bacterial invasion of epithelial cells since cementum is the tissue most often affected in peripheral caries.^41^ MMP9, a matrix‐remodelling enzyme, was also uniquely identified in cementum and is essential for cementoblast‐mediated ECM turnover.^42^, ^43^ Similarly, interferon‐induced transmembrane protein 5 (IFITM5) and IGF1, both linked to osteogenic mineralisation,^44^, ^45^ were found only in cementum, highlighting its bone‐like yet functionally distinct role. Notably, serine protease inhibitor 2 (SERPINE)^46^ and thrombospondin 2/3 (THBS2/THBS3)^47^ regulate ECM integrity and were unique to cementum, suggesting a well‐regulated but dynamic matrix environment.

Clustering analyses assessed global similarities and differences in protein abundance between samples. These methods group samples based on shared variance, helping to visualise underlying biological patterns.^48^ Interestingly, the degree of clustering appeared to correlate with the tissue's ability for remodelling. Not all dental tissues show a clear ageing or disease pattern at the protein level. Dental pulp displayed minimal variability in mature samples, likely due to its protected environment and limited adaptability early in life. Variability increased in older and diseased samples, suggesting dynamic tissues like pulp mount broader proteomic responses under stress, reflecting both repair and susceptibility to dysregulation. In contrast, hard tissues showed less dramatic shifts, consistent with the stability of mineralised tissues, which are less metabolically active and less prone to proteomic change. In hard tissues, disease‐related changes may reflect loss or degradation rather than active remodelling, as has been previously stated with dental tissues.^49^, ^50^

In enamel, there were no statistically significant differences in the abundance of proteins with ageing. Ameloblasts undergo apoptosis during amelogenesis or die following enamel mineralisation, leaving an acellular mature enamel containing minor amounts of organic matter interspersed between enamel crystals.^51^ In horses, the enamel production and mineralisation process is thought to be completed by 5 years of age for all molars,^52^ though others report enamel deposition for up to 5 years post eruption.^9^, ^53^, ^54^ Therefore, it is unsurprising that the proteome of the enamel remained unaffected by ageing. As this is the first study to characterise the proteomic profile of enamel during ageing in any species, direct comparisons with existing literature are not available. However, in the long‐lived *Equus hemionus*, extended enamel deposition into the root was identified, and it has been hypothesised that enamel deposition is prolonged in this species.^55^ It is therefore possible that the age of completion of enamel mineralisation in horses is heterogeneous.

Differential abundance analysis of the cementum revealed a dysregulation of proteins associated with cementogenesis and mineralisation with ageing. More specifically, an increase in the abundance of negative regulators of mineralisation matrix gla protein (MGP), CALR and fibroblast growth factor 23 (FGF23) was noted.^56^, ^57^, ^58^ Interestingly, FGF23 is regulated by PHEX and the reduction in PHEX abundance observed in enamel may lead to the upregulation of FGF23 in cementum following crosstalk between the two tissues.^59^ Conversely, ALPL which promotes mineralisation was also increased in ageing, alluding to the complex regulation of the ECM in dental tissues.^60^ SPP2 was also increased, which can lead to a reduction in the production of cementum and the differentiation of cementoblasts through limiting the bioavailability of BMP2.^61^, ^62^, ^63^, ^64^, ^65^ Matrix remodelling also appeared to occur in the ageing cementum with an upregulation in COL12A1, which is associated with fibrosis and scar formation instead of regeneration, and in the matrix metalloproteinases MMP2 and MMP13, which in addition participate in the ageing‐related senescence‐associated secretory phenotype,^66^, ^67^, ^68^ inflammation^69^ and oxidative stress.^70^ This finding suggests an ongoing or heightened catabolic activity in the ageing cementum, potentially reflecting continuous turnover, repair processes, or early signs of age‐associated pathological remodelling. This is further supported by the reduction of cellular proteins in older cementum including AHCY,^71^ CCT^72^ and FAM171B.^73^ The decline of these predominantly intracellular and metabolically active proteins indicates a shift to a more matrix‐dominated, potentially dysregulated state, which is a common hallmark of ageing in connective tissues.^74^ However, IGFBP5 and SFRP2 which induce cementogenesis and tissue regeneration were also increased^75^, ^76^ highlighting that despite ageing and the observed age‐related matrix dysregulation in cementum, healthy aged cheek teeth might retain some capacity for regeneration.

Interestingly, although fewer overall protein differences were observed in dentin compared to cementum, a similar pattern was seen regarding the proteomic shifts associated with ageing in dentin. An age‐related reduction in the abundance of proteins participating in dentinogenesis including DSPP, MDK, PTN, semaphorin‐3E (SEMA3E) and ANXA6 was noted. More specifically, DSPP, the most abundant non‐collagenous protein in dentin, and ANXA6 participate in dentin mineralisation.^77^, ^78^, ^79^ Whereas SEMA3E and the heparin‐binding growth factors MDK and PTN promote the differentiation of odontoblasts and formation of dentin while PTN additionally protects dental pulp stem cells from senescence.^80^, ^81^, ^82^ The reduction in the abundance of these proteins indicates a similar age‐related trajectory to that evident in cementum. This common decline suggests a proteome characteristic of cellular growth, proliferation and a youthful developmental environment is progressively lost with age. Taken together, these age‐related alterations indicate the production and quality of secondary and tertiary dentin which protect the dental pulp from exposure and inflammation may be compromised in ageing which might explain the higher prevalence of dental pathologies such as occlusal fissures and infundibular caries in older horses.^9^, ^83^

Dental pulp showed the greatest variability, yet differential abundance between mature and old pulp revealed fewer changes than cementum or dentin, despite more proteins being identified in pulp. This may be attributed to both biological and statistical factors. Biologically, pulp is a highly cellular and vascularised tissue with a diverse and dynamic proteome, which may be buffered against ageing effects due to active homeostatic regulation. It is possible that the dental pulp is more resilient and less affected by ageing compared to the other dental tissues partly retaining its homeostasis and ability for regeneration. Similarly to old cementum, MGP and SPP2 were increased in the dental pulp with ageing, suggesting hypomineralisation of dentin and possibly inhibition of the BMP2‐induced odontogenic differentiation of dental pulp cells.^84^, ^85^ In addition, aggrecan (ACAN) was reduced in pulp in ageing suggesting a degradation or diminished synthesis of key structural components of the pulp's ECM.^86^ Furthermore, among the differentially abundant proteins in the aged dental pulp were ACSF2 and DDX23 which are associated with ageing in other tissues through mitochondrial dysfunction and guarding of genomic stability, respectively.^87^, ^88^ In contrast, cementum is more mineralised and acellular, with a simpler proteome that may be more directly exposed to cumulative age‐related damage, making the few proteins present more susceptible to detectable changes. Statistically, the larger number of proteins in pulp increases the multiple testing burden, making it harder for individual proteins to pass the FDR threshold. Meanwhile, the smaller protein set in cementum faced less stringent correction, allowing a higher proportion to reach significance. Additionally, lower biological variability in cementum may enhance the signal‐to‐noise ratio, further contributing to the detection of significant age‐related changes despite the smaller proteome.

No significant differentially abundant proteins (at the adjusted *p*‐value) were found as a result of disease for all sampled dental tissues apart from COL15A1 in cementum. Carious lesions, the prevailing pathology for the diseased cheek teeth in the present study, involve more often the cementum and only Grade 2 and above lesions will extend beyond the cementum into the enamel whereas Grade 3 lesions will affect the dentin.^41^ It is therefore possible that disease‐related alterations in the proteome of enamel, dentin and pulp were not found due to the severity of the pathology. The exact role of COL15A1 in cementum and disease remains unclear, however, its dysregulation has been linked to periodontitis.^89^ Of note, pathway analysis predicted the inhibition of the pathway ‘Regulation of IGF transport and uptake by IGFBPs’ in diseased pulp and the cementum of the diseased cheek teeth showed a non‐significant reduction in IGF1 and IGFBP5 in comparison to the age‐matched clinically healthy teeth. IGFBP5 and IGF1 induce cementogenesis and IGFBP5 was found to improve tissue regeneration in an in vitro model of periodontal disease,^75^, ^90^ suggesting that the ability for some regeneration which is retained in the aged cementum might be lost in disease. Other pathways predicted to be inhibited in diseased pulp also related to the ECM, such as the pathways ‘ECM organisation’, ‘Collagen biosynthesis and modifying enzymes’ and ‘Wound healing signalling pathway’. This reduction points towards an impaired capacity for effective tissue repair in disease. In contrast, pathways predicted to be upregulated in dental pulp were associated with cellular processes, for example, RAR (retinoic acid receptor) activation and sheddase activity. RAR signalling is broadly involved in cell differentiation^91^ and tissue development,^92^ and its increase in disease could imply dysregulated attempts at repair or altered cellular states. Increased sheddase activity, often plays a role in regulating cell–cell and cell‐matrix interactions, and its elevation may contribute to altered cellular communication and release of signalling molecules in the inflamed/diseased pulp.^93^, ^94^ A limitation in the determination of disease‐related alterations in the proteomic profile of dental tissues in the present study is the heterogeneity in the type, severity and duration of the dental pathology of the diseased cheek teeth.

Limitations include small sample size, restricted validation due to limited equine‐specific reagents, and heterogeneity in disease samples. Despite these constraints, findings provide a foundation for future studies, with targeted validation approaches (e.g., multiple reaction monitoring) to enhance translational potential.

Analysis of overlapping differentially abundant proteins across dental tissues revealed both shared and tissue‐specific responses to ageing and disease. The relatively small number of proteins commonly increased across tissues in ageing suggests that, while each tissue may follow its own ageing trajectory, certain molecular pathways are consistently altered. Ageing and disease affect dental tissues differently but share molecular changes, particularly in ECM integrity and immune signalling. Limited overlap across tissues highlights process complexity and tissue specificity, yet consistently altered proteins may offer therapeutic targets for oral degeneration.

## 5 CONCLUSIONS

Ageing significantly impacts the ECM across all dental hard tissues, indicating reduced quality that may compromise mechanical properties and resistance to trauma, microbes and disease. Proteins associated with regenerative capacity declined with age but persisted in cementum and pulp. In diseased cheek teeth versus age‐matched healthy teeth, few differentially abundant proteins were detected, likely due to sample heterogeneity. Both ageing and disease disrupt ECM homeostasis and integrity, though with tissue‐specific patterns and limited overlap. Our results establish a baseline proteomic map of equine dental tissues, identifying specific protein candidates that warrant further functional study to determine their role in the progression of dental pathology in older horses.

## FUNDING INFORMATION

This work was funded by The Horse Trust Small Grant G2029. Anders Jensen was supported by a Dunhill Medical Trust PhD studentship.

## CONFLICT OF INTEREST STATEMENT

The authors declare no conflicts of interest.

## AUTHOR CONTRIBUTIONS

**Anders Jensen:** Conceptualization; methodology; formal analysis; investigation; data curation; writing – original draft; writing – review and editing; visualization. **Danae Emilie Zambouli:** Formal analysis; data curation; writing – original draft; writing – review and editing; visualization. **Iris Gringel:** Formal analysis; investigation; writing – review and editing. **Zoe Nugent:** Investigation; writing – review and editing. **Kazuhiro Yamamoto:** Funding acquisition; writing – review and editing. **Lee Cooper:** Methodology; investigation; writing – review and editing. **Andrew J. Peffers:** Conceptualization; methodology; writing – review and editing. **Guido Rocchigiani:** Methodology; writing – review and editing. **Mandy J. Peffers:** Conceptualization; methodology; formal analysis; investigation; data curation; writing – original draft; funding acquisition; writing – review and editing.

## DATA INTEGRITY STATEMENT

Mandy J. Peffers had full access to all the data in the study and takes responsibility for the integrity of the data and the accuracy of data analysis.

## ETHICAL ANIMAL RESEARCH

This project was approved by an ethical committee of the University of Liverpool, Institute of Infection Veterinary and Ecological Sciences (ethics no: VREC1171).

## INFORMED CONSENT

Informed consent was obtained for all samples taken from horses that underwent surgery.

## Supporting information


**Figure S1.** Functional roles of identified proteins in dentin using clusterProfiler R.

**Figure S2.** PCA.

**Table S1.** Table of tooth details for the study.

**Table S2.** Top 30 DA proteins across age and disease cementum (Excel).

**Table S3.** Top 30 DA proteins across age and disease dentin (Excel).

**Table S4.** Top 30 DA proteins across age and disease enamel (Excel).

**Table S5.** Top 30 DA proteins across age and disease pulp (Excel).

## Data Availability

The data that support the findings of this study are openly available in the PRIDE submission database under the following identifiers: Enamel ‐ https://www.ebi.ac.uk/pride/archive/projects/PXD065888, Dentin ‐ https://www.ebi.ac.uk/pride/archive/projects/PXD065894, Cementum ‐ https://www.ebi.ac.uk/pride/archive/projects/PXD066267, and Pulp ‐ https://www.ebi.ac.uk/pride/archive/projects/PXD067261.
