# Supplementary material for: Proteomic signatures of equine dental tooth tissues in ageing and disease
Source: Equine Vet J. 2026 May 15;58(6):1552–69. doi: 10.1002/evj.70167 (PMC13657193; doi:10.1002/evj.70167)

Equine Veterinary Journal BEVA

Figure S2:

Clustering analysis of cementum. A. Principal component analysis plot of the first two principal components. Biological groups are represented by different colours; mature adult = blue, Old = purple and Diseased = purple. B. Dendrograms generated using hierarchical clustering (Ward's method, Euclidean distance) illustrating the intrinsic similarities between tissues. Sample labels are aligned vertically, and clustering reflects condition-specific protein abundance patterns. Figures were made using ggplot2 R package (version 3.5.2)

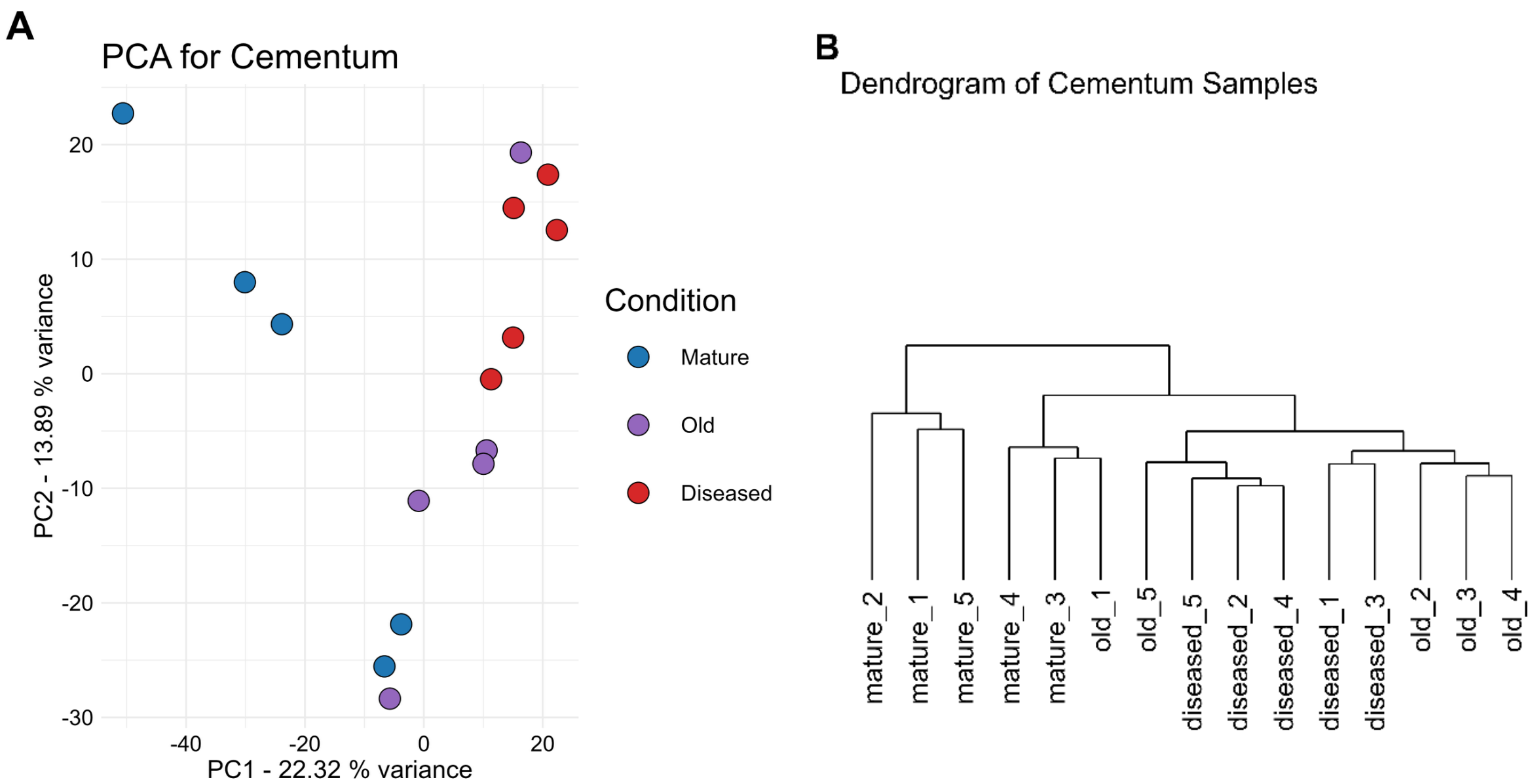

Clustering analysis of dentin. A. Principal component analysis plot of the first two principal components. Biological groups are represented by different colours; mature adult = blue, Old = purple and Diseased = purple. B. Corresponding dendrograms generated using hierarchical clustering (Ward's method, Euclidean distance) illustrating the intrinsic similarities between dentin samples. Sample labels are aligned vertically, and clustering reflects condition-specific protein abundance patterns. Figures were made using ggplot2 R package (version 3.5.2).

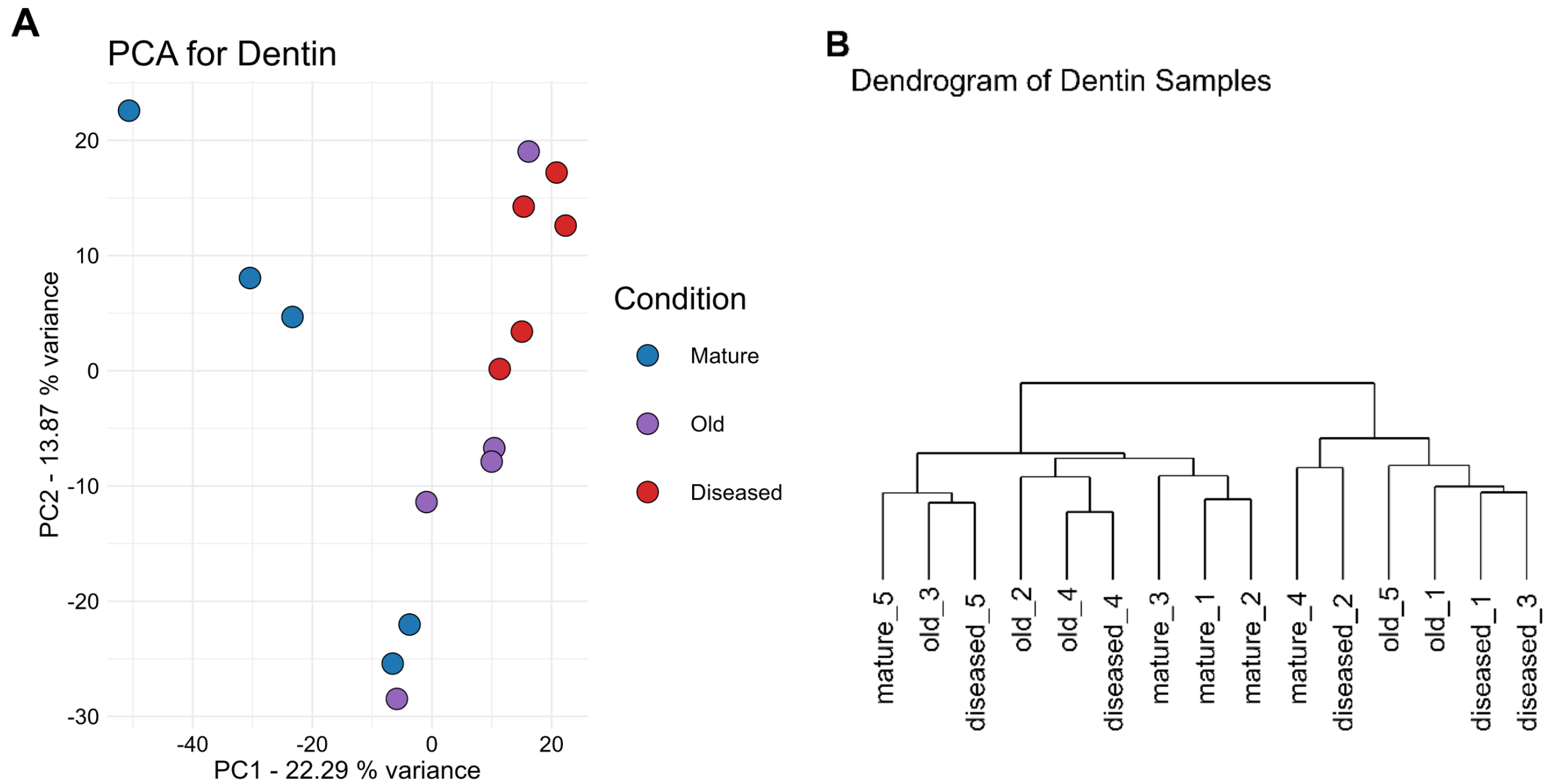

Clustering analysis of enamel. A. Principal component analysis plot of the first two principal components. Biological groups are represented by different colours; mature adult = blue, Old = purple and Diseased = purple. B. Corresponding dendrograms generated using hierarchical clustering (Ward's method, Euclidean distance) illustrating the intrinsic similarities between enamel samples. Sample labels are aligned vertically, and clustering reflects condition-specific protein abundance patterns. Figures were made using ggplot2 R package (version 3.5.2

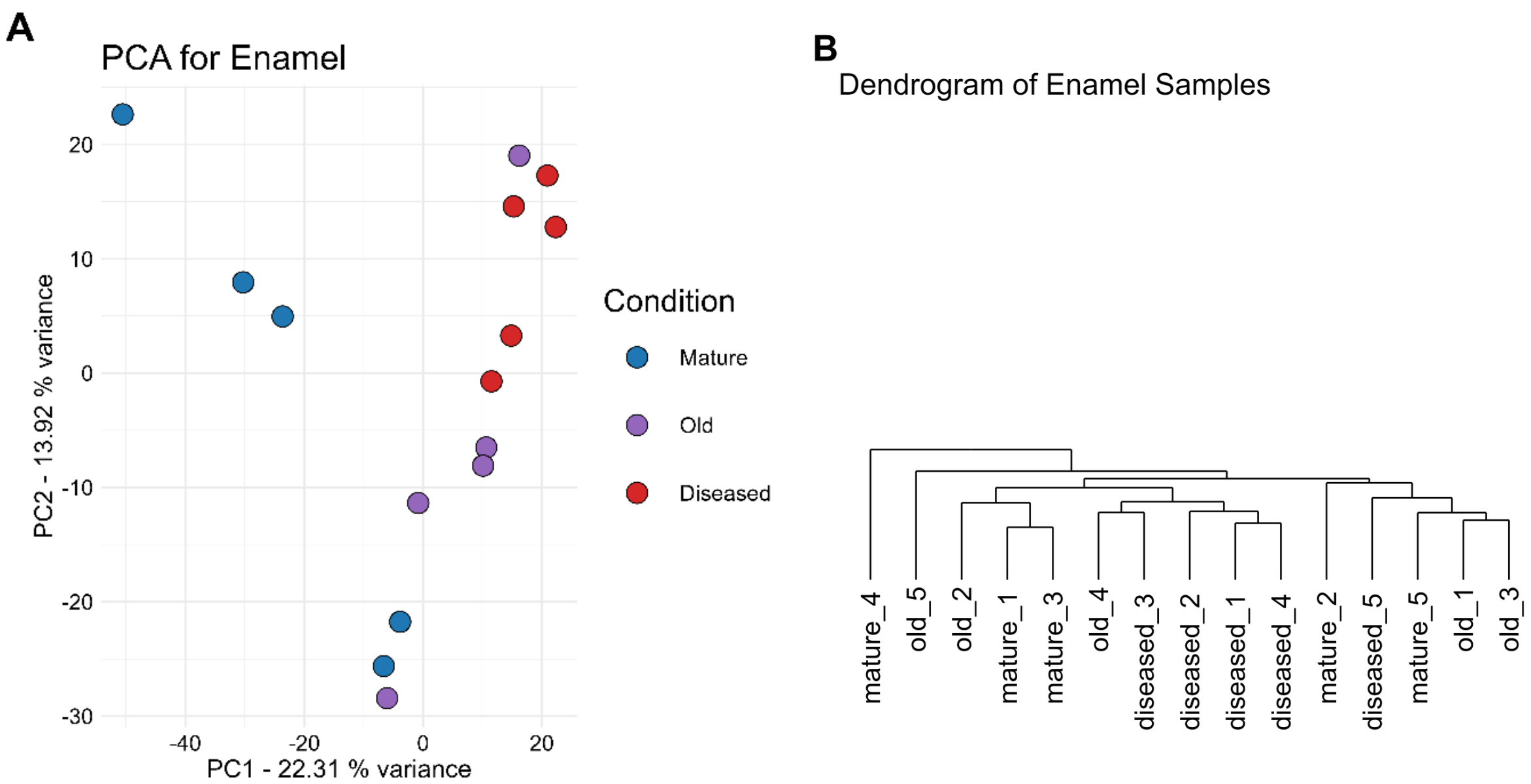

Clustering analysis of dental pulp. A. Principal component analysis plot of the first two principal components. Biological groups are represented by different colours; mature adult = blue, Old = purple and Diseased = purple. B. Corresponding dendrograms generated using hierarchical clustering (Ward's method, Euclidean distance) illustrating the intrinsic similarities between pulp samples. Sample labels are aligned vertically, and clustering reflects condition-specific protein abundance patterns. Figures were made using ggplot2 R package (version 3.5.2).

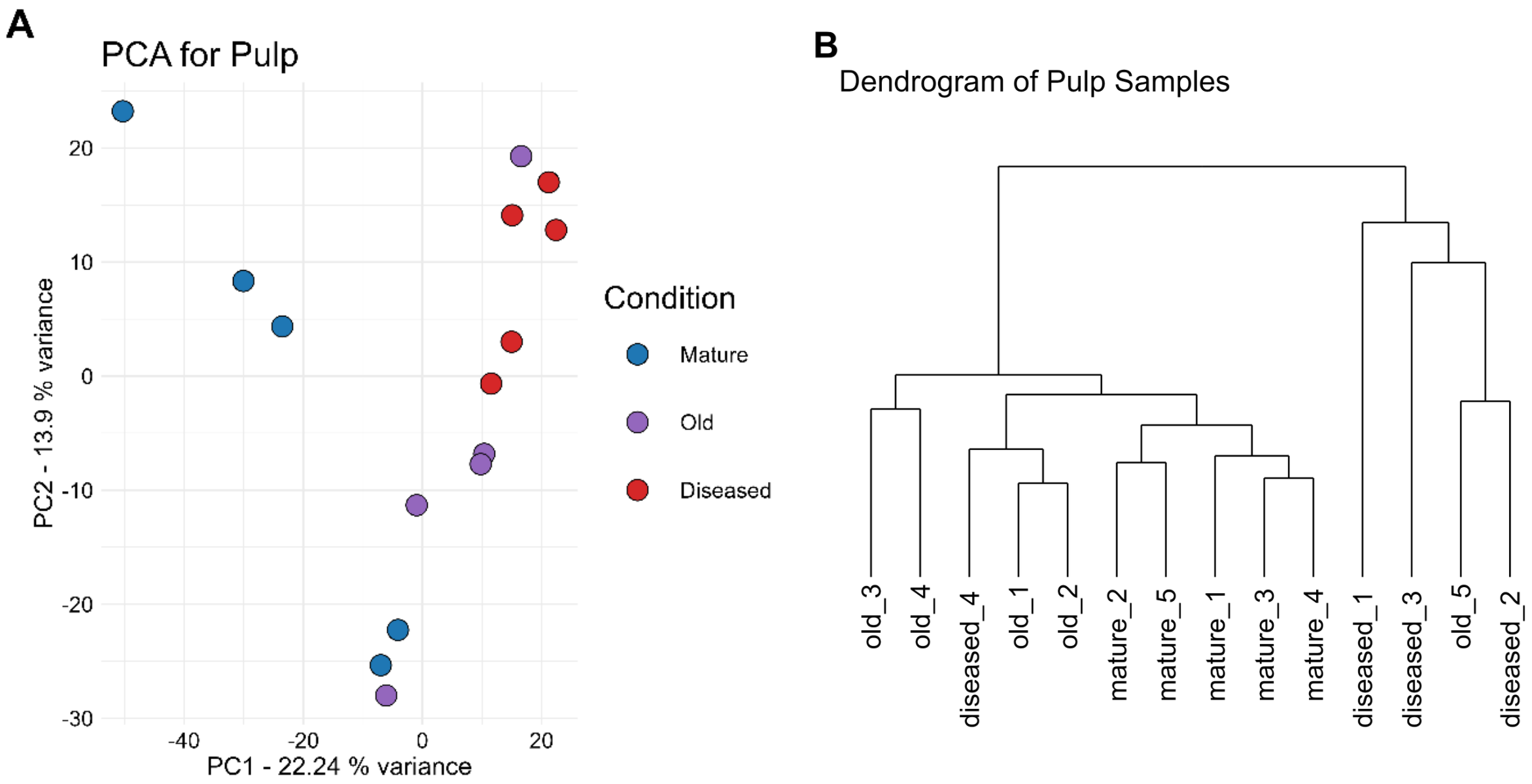

Supplement: Supplementary file 1 [file EVJ-58-1552-s002.pdf]
