# Supplementary material for: Proteomic signatures of equine dental tooth tissues in ageing and disease
Source: Equine Vet J. 2026 May 15;58(6):1552–69. doi: 10.1002/evj.70167 (PMC13657193; doi:10.1002/evj.70167)

Equine Veterinary Journal

BEVA

Figure S1: Functional roles of unique pulp proteins using gene ontology enrichment analysis. Dot plots display significantly enriched GO terms: A. Biological Process (BP), B. Molecular Function (MF), and C. Cellular Component (CC). The size of each dot represents the number of proteins annotated to the respective GO term (Count), while the colour gradient indicates the adjusted p-value (p.adjust), with red representing higher significance. Figures were produced using the clusterProfiler R package (version 4.6.2).

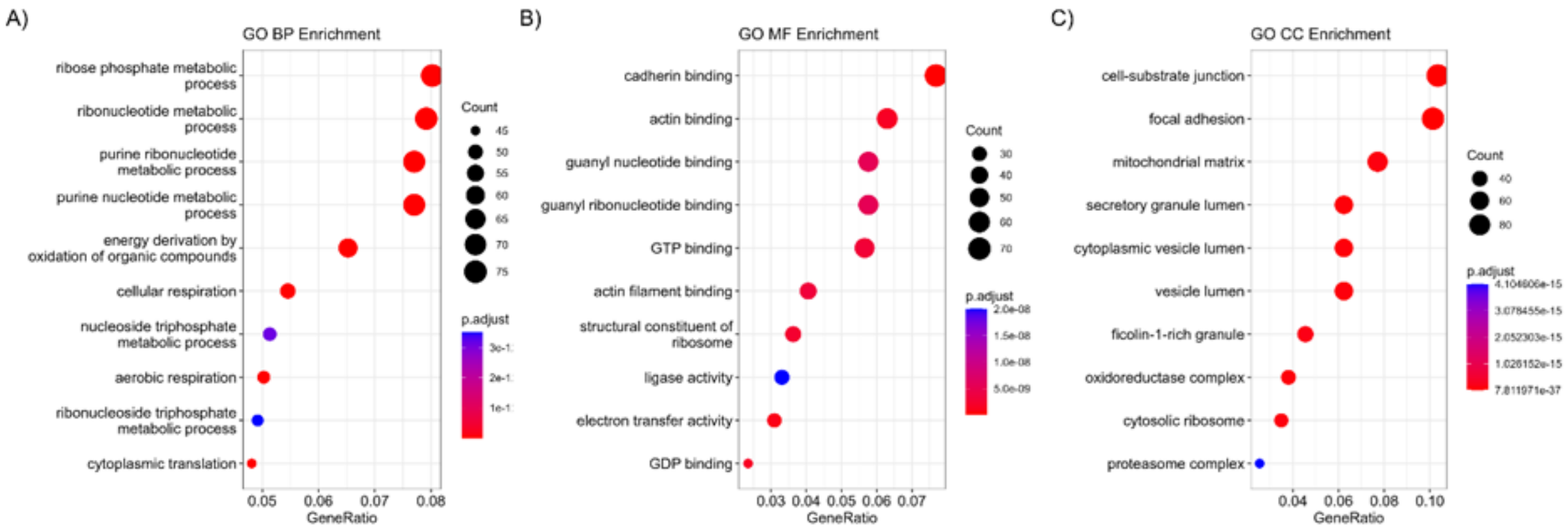

Supplement: Supplementary file 2 [file EVJ-58-1552-s003.pdf]
