# Supplementary material for: Proteomic signatures of equine dental tooth tissues in ageing and disease
Source: Equine Vet J. 2026 May 15;58(6):1552–69. doi: 10.1002/evj.70167 (PMC13657193; doi:10.1002/evj.70167)
Supplement: Supplementary file 3 [file EVJ-58-1552-s006.pdf]

Equine Veterinary Journal BEVA

Table S1: Table of tooth details for the study.

Group	Age (years)	Source	Tooth
young	6	mandible	09
young	6	maxilla	09
young	7	maxilla	06
young	9	mandible	09
young	7	maxilla	06
old	19	mandible	06
old	17	mandible	06
old	19	maxilla	09
old	20	mandible	09
old	24	maxilla	09
diseased	24	mandible	06
diseased	20	maxilla	06
diseased	19	maxilla	09
diseased	18	mandible	09
diseased	18	maxilla	06
